# Allometric Trajectories and “Stress”: A Quantitative Approach

**DOI:** 10.3389/fpls.2016.01681

**Published:** 2016-11-09

**Authors:** Tommaso Anfodillo, Giai Petit, Frank Sterck, Silvia Lechthaler, Mark E. Olson

**Affiliations:** ^1^Dipartimento Territorio e Sistemi Agro-Forestali, Università degli Studi di PadovaLegnaro, Italy; ^2^Forest Ecology and Forest Management Group, Wageningen UniversityWageningen, Netherlands; ^3^Instituto de Biologia, Universidad Nacional Autonoma de MexicoMéxico, México

**Keywords:** fitness, scaling, morphospace, operationalization, plasticity

## Abstract

The term “stress” is an important but vague term in plant biology. We show situations in which thinking in terms of “stress” is profitably replaced by quantifying distance from functionally optimal scaling relationships between plant parts. These relationships include, for example, the often-cited one between leaf area and sapwood area, which presumably reflects mutual dependence between sources and sink tissues and which scales positively within individuals and across species. These relationships seem to be so basic to plant functioning that they are favored by selection across nearly all plant lineages. Within a species or population, individuals that are far from the common scaling patterns are thus expected to perform negatively. For instance, “too little” leaf area (e.g., due to herbivory or disease) per unit of active stem mass would be expected to incur to low carbon income per respiratory cost and thus lead to lower growth. We present a framework that allows quantitative study of phenomena traditionally assigned to “stress,” without need for recourse to this term. Our approach contrasts with traditional approaches for studying “stress,” e.g., revealing that small “stressed” plants likely are in fact well suited to local conditions. We thus offer a quantitative perspective to the study of phenomena often referred to under such terms as “stress,” plasticity, adaptation, and acclimation.

## Introduction

Like many terms in plant ecology, the term “stress” is both very important and vague. Authors have debated its definition in various contexts for decades (e.g., Levitt, 1972) and this debate continues (e.g., Körner, 2003; Lortie et al., 2004). Because the term is so important, it would be useful to operationalize it, to make it readily accessible to empirical study. One of the pitfalls of such operationalization is that definitions can represent simply artificial categories rather than true natural phenomena (see for example discussions of efforts to operationalize “adaptive radiation” Olson and Arroyo-Santos, 2009). We focus on certain situations that are traditionally discussed in terms of “stress” but in which the term is not only unnecessary but might actually be hiding important adaptive phenomena.

Our approach builds on the observation that many plant attributes covary with one another in highly predictable ways, that is, plants grow allometrically (or isometrically). Plant ecologists document webs of trait covariation that seem to involve most crucial plant traits (Weiner, 2004; Westoby and Wright, 2006). For example, wood traits such as density, branch and stem dimensions, and mechanical resistance to bending are tightly correlated (Sterck et al., 2006a; Rosell et al., 2012; Castorena et al., 2015). Leaf traits such as leaf lifespan, leaf mass, photosynthetic capacity and respiration are also closely coupled as described by the leaf economic spectrum (e.g., Reich et al., 1997; Sterck et al., 2006b). At the whole plant level, the area and mass scaling relations between organs such as leaves and stem and their tissues (e.g., xylem, phloem) are under strong selection (e.g., Cannell and Dewar, 1994; Zhang et al., 2016). Many of these patterns of covariation seem to reflect evolutionarily optimal relationships, i.e., not global optima for any one trait but the “least bad” combination possible given their conflicting demands (e.g., Niklas, 1994; West et al., 1999; Niklas and Enquist, 2001). These relationships, manifest in stable allometric trajectories, are largely thought to be maintained by natural selection. In other words, some combinations are possible but usually not favored by selection. For example, plants with dense wood usually bear small leaves but plants with low-density wood bear large ones (Olson et al., 2009). Presumably the combination of high density wood and very large leaves is one not generally favored. These two observations—that plant traits frequently covary, and that these relationships can vary to some degree—motivate our proposed means of studying phenomena traditionally referred to as “stress.”

The central prediction of our proposal is that distance from allometric scaling lines should be associated with differences in performance or fitness. There is no need for recourse to the term “stress” at all in making this formulation. Performance here is understood as any index that should affect fitness, e.g., photosynthetic efficiency, mechanical support, hydraulic resistance, etc. Fitness is understood to comprise its three components, survivorship, mating success, and fecundity. If an allometric relationship, e.g., leaf mass *vs.* stem volume, is maintained within a species by selection, then a drastic displacement from the relationship is expected to result in lower performance. For example, sustained defoliation markedly reduces fitness (Anderegg and Callaway, 2012). Drastic removal of sapwood tissue can have a similar effect. Both of these disturbances result in marked movement into spaces distant from the common allometric scaling slope. We propose that distance from the line should be associated with quantifiable differences in performance, and this quantifiability obviates the need for categorizing a given individual as “stressed” or not. We will also show how this approach can reveal situations in which responses to “stress” are in fact adaptive. A key element in generating predictions and interpreting patterns under our approach is a theoretical understanding of allometric relationships.

## Theoretical Understanding of Allometric Trajectories

Numerous theoretical considerations underpin the understanding of allometric relationships, as reflected in evolutionary optimality models (e.g., Banavar et al., 1999; West et al., 1999; Enquist and Niklas, 2002; Sterck and Schieving, 2007; Dewar et al., 2009). These models have as central tenets that organisms have three fundamental components: a volume of metabolically active cells, resource distribution networks, and metabolite exchange surfaces (Banavar et al., 2014). In other words, allometric relationships between traits reflect evolutionary convergence on the “best” combination of investment in the three components. One of the most studied allometric scaling patterns is the one between metabolic activity (*B*) and body mass (*M*), the exponent of which is clearly 3/4 in animals (Kleiber, 1932), whereas for plants it is generally between 1 and 3/4, depending on how much dead tissue (heartwood) makes up the “body mass” (Reich et al., 2006; Mori et al., 2010). Based on these fundamental relationships, other predictions can be generated. For example, in the simplest case of a tree whose crown shape remains constant as the tree grows larger, *M* should scale *vs.* tree height (*h*) as *M* ∝ *h*^4^, *B* ∝*h*^3^ and leaf area ∝*h*^3^ (e.g., Simini et al., 2010). These examples illustrate that these relationships are widespread and span many species. They also help identify situations of interest when plants deviate from predicted relationships.

**FIGURE 1:**
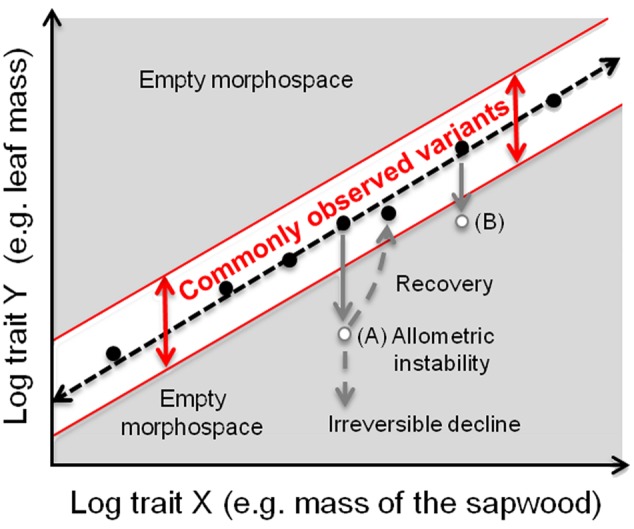
**How allometric trajectories can be used for quantitatively studying phenomena traditionally discussed in the context of “stress.”** The simplest case is to consider a single species in a specific environment. Most individuals of a tree species have the typical proportionalities between traits (e.g., log Y = leaf mass and log X = mass of the sapwood), with some variation about this line frequently observed (white area), e.g., as heritable variants in natural populations or responses to differences in local conditions. Outside of this area, a wide “empty morphospace” (in gray) (*sensu* Olson, 2012) is potentially available for different morphotypes with allocation patterns that deviate from the commonly observed variants. Individuals in these areas are expected to have lower performance or fitness relative to those within the white zone. The prediction that distance from the white zone broadly correlates negatively with performance is readily testable. A tree at point A would be expected to have lower performance/fitness than a tree within the common morphospace (white band). For example, higher respiratory costs correlated with a larger body biomass per unit of leaf would decrease individual performance. Therefore, plants would be expected to recover the optimal trait combination. At some threshold level of damage, they presumably cannot recover (irreversible decline) and die. Selection is therefore expected not to favor variants that lie in empty morphospace, such as a tree in points A or B. Allometric trajectories with different intercepts within the white area likely represent different trait proportionalities favored in different environmental conditions, e.g., lower intercepts in resource-rich sites, indicating that a unit of leaf area supports a higher amount of consuming/supporting tissues because annual carbon gain (i.e., assimilation) is higher (see **Figure 2B** and text for further explanations).

These theoretical considerations motivate the fundamental prediction of our approach, which is that distance from the allometric slope should be associated with variation in performance or fitness (**Figure 1**). Many studies have shown that variants that fall far from common allometric scaling slopes have lower performance or fitness than individuals that fall close to the line (cf. Sinervo and Huey, 1990; Sinervo and Licht, 1991; Bertram et al., 2009). For example, in *Raphanus raphinastrum*, corolla tube-stamen length poportions, which are constant across most *Brassicaceae*, was readily altered in just a few generations of artificial selection (Conner et al., 2011). Similar results are found in animal studies as well: butterflies with relatively large fore- or hind- wings had much lower reproductive success than conspecifics with wild-type hind- and fore- wing proportionality (Frankino et al., 2007). These results show that variants corresponding to “empty” morphospace (gray areas in **Figure 1**) can be readily produced. That they are only rarely observed in nature strongly suggests that they are eliminated by selection, favoring instead those in the white band in **Figure 1**). We will show how empirical allometric relations can help to examine phenomena traditionally referred to as “stress” in terms of departure from common allometric scaling relationships. We provide examples of two situations commonly discussed in the context of “stress,” plants affected by defoliation (which when sustained leads to lowered fitness) and plants exposed to different environmental growth conditions (in which, on the contrary, plants show adaptive responses, i.e., maximal fitness *in that environment*).

## Reversible Defoliation

**FIGURE 2:**
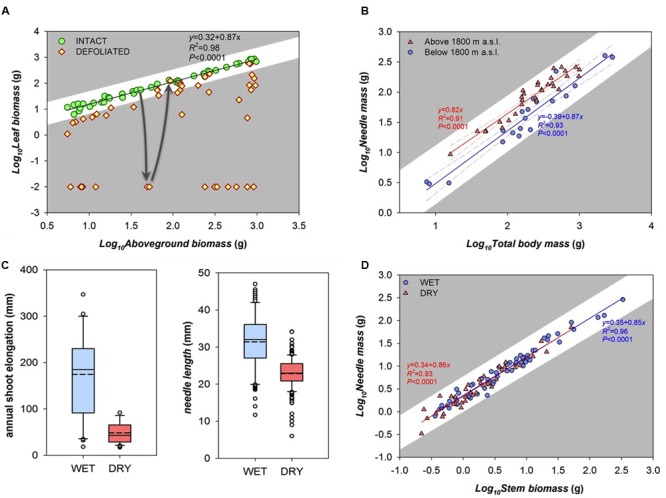
**Empirical relationships between traits and different notions of “stress.” (A)** Defoliation in *Moringa oleifera* trees. As in most plants, undamaged shoots have a highly predictable relationship between stem mass and leaf mass (circles). Leaf harvesting temporarily diverts this relationship (diamonds), but plants stripped of their leaves sooner or later recover the pre-damage leaf mass *vs.* stem mass relationship (arrows). **(B–D)** Examples of “stressful” environments. **(B)** Possible variants at different elevations: in *Pinus cembra* from below (circles) *vs*. above (triangles) 1800 m a.s.l., the scaling of leaf mass *vs.* total body mass (roots included) follows the same exponent (∼0.85), but leaf mass per unit of body mass (i.e., Y intercept) is higher in high elevation trees. **(C)** Boxplot of annual shoot growth and needle length between wet (“favorable”) and dry (“stressful”) sites in *Pinus sylvestris* (dashed and solid lines are mean and median values respectively). This approach seems to show categorical differences between trees in sites that could be arbitrarily classified as stressed and unstressed. **(D)** However, when the same samples of **Figure 2C** are plotted as part of an allometric series, it is clear that the scaling of leaf mass *vs.* shoot mass (the last three growing years) converges on the same trajectory in both wet (circles) and dry (triangles) conditions. This result highlights that the species is able to build similar allometries of the distal parts of the plant in spite of different environments.

Our first example is one in which “stress” results in reversible, quantitative deviations from local optima. For example, leaf and stem mass covary in a highly predictable fashion in all leaf-bearing species studied so far (Enquist and Niklas, 2002). Here, we present leaf mass *vs.* stem mass data from young shoots of coppiced individuals of the tropical tree *Moringa oleifera*. Note in what follows how these patterns can be discussed with reference to the specific selection pressures with no need to refer to generic and vague notions of “stress.” In intact shoots, leaf mass scales with stem mass in a highly predictable way (**Figure 2A**; the exponent of the allometric relationship is 0.87). These trees are located in a public area in a village, where local people regularly harvest the leaves for consumption. Sometimes they strip all of the leaves off of a shoot, leaving only the stem, or most of the leaves, leaving only the oldest leaves, and sometimes they remove only the tenderest terminal leaves. The leaf mass *vs.* stem mass relationships of these “defoliated” shoots are also shown in **Figure 2A**. Over time, the plants produce new leaves both from axial as well as terminal buds and recuperate the leaf mass *vs.* stem mass relation of undamaged shoots (arrows in **Figure 2A**).

This baseline leaf mass *vs.* stem mass relationship is thought to be one favored by natural selection, and this expectation leads to testable predictions regarding “stress” understood as deviation from the baseline allometric relationship. The leaf mass *vs.* stem mass relationship is thought to be driven by the mutual metabolic relationship between leaves, which produce photosynthates, and stems, which consume photosynthates and mechanically support leaves and supply them with water. Defoliation moves trees away from this relationship, and potentially decreases their performance. In a similar way, shoots with substantial amounts of stem tissue removed would lose significant amounts of water conducting and nutrient storage volume. Although defoliation experiments show a variety of short term responses to tissue removal (Ferraro and Oesterheld, 2002), both loss of stem as well as loss of leaves are expected on average and in the long run to result in lower net photosynthesis and lowered fitness components (Anderegg and Callaway, 2012) such as seed production (i.e., fecundity). Irreversible damage presumably marks the amount of damage to one or both variables that leads to death. This view allows empirical investigation of the degree to which distance from allometric trajectories is associated with quantifiable differences in performance. Reference to “stress” would provide absolutely no empirical advantage or theoretical insight. We now turn to another situation in which the word “stress” is commonly used, and again we show that our allometric alternative provides a much more constructive perspective, helping highlight that plants can adjust their structure in different conditions of resource availability, maximizing the fitness possibilities of each environment.

## Allometry and Growth in “Stressful Environments”

A very common use of the word “stress” in plant ecology is to refer to environments that limit growth and are therefore “stressful.” These examples highlight notions of “stress” as lowered productivity, obvious inheritance from agricultural settings, where lowered productivity is unwelcome. Such value-laden terminology has no place in science, as our examples will illustrate. Our first example comes from trees growing at treeline, which are traditionally regarded as “limited” or “stressed” because of their slower growth and irregular crown morphologies, a pointless and value-laden classification. From an allometric perspective, however, stone pine (*Pinus cembra*) trees growing at high elevation (above 1800 m a.s.l.) have similar needle mass *vs.* body mass scaling slopes but different Y intercepts as compared to trees from lower elevation (**Figure 2B**). Similar slope means that the crucial trait relationships are maintained in spite of different climate conditions and crown shapes. Higher leaf area for a given body mass in treeline trees might be interpreted as a compensation for the lower annual assimilation per needle mass due to the shorter growing season. Thus a higher leaf area is needed to sustain the respiratory C-losses that likely scale isometrically with body mass at any elevation (Reich et al., 2006; Mori et al., 2010) confirming that different allocation strategies (i.e., amount of leaves per unit of body mass) are possible under the same scaling relationship (i.e., the relative co-variation of both traits). This example shows that our approach highlights important biological questions masked by the traditional categorical approach.

Our second example comes from Scots pine (*Pinus sylvestris*) trees (**Figures 2C,D**), which grow tall on deep soils but are short and thin, on shallow, rocky soils. In terms of traditional value laden terminology, the “stunted” trees on rocky soils are often described as growing in “stressful” conditions, as reflected by their much lower annual length growth increments and shorter needles as compared to taller trees on moist, deep soils (**Figure 2C**). Our allometric approach highlights that the often value-laden terminology of “stress” in fact hides much valuable biological insight, even leading to very different conclusions. Plotting nearly the same variables against one another (**Figure 2D**) shows that, rather than two distinct categories, the plants considered “stressed” and “unstressed” are in fact indistinguishable with regard to their patterns of trait covariation. In this example, needle mass scales with stem biomass in exactly the same way in “stressed” and “unstressed” plants. This result highlights an entirely different set of biological issues as opposed to the traditional categorical, value-laden approach. Whereas the categorical approach highlights limitations of growth on dry sites as opposed to imaginary optima, the allometric approach instead shows that the plants in both situations are constructing the distal part of the stem (that bears the needles) along essentially identical allometric scaling relationships, though of different sizes.

These examples illustrate how, from an allometric perspective, the notion of “stress” is largely an inheritance from forestry and agriculture, in which any factor that reduces yield is described with negative terminology (Körner, 2003). However, from an evolutionary point of view, it is hard to see how the small trees of dry sites can be classified as “suboptimal,” “limited,” or “stressed.” Instead, their small stature likely represents an adaptive response to prevailing conditions. That they scale similarly to their larger conspecifics on deep, moist soil in their trait relationships gives no reason to consider them as “stressed,” an observation that the traditional categorical approach conceals.

## Conclusion

Our quantitative approach does not require arbitrary categorizations of “stress,” because it involves testing the prediction that distance from the general allometric slope should be associated with differences in performance (**Figure 1**). From this point of view, the dividing area between adaptive differences, which should maximize performance in the relevant environment, and those that push individuals beyond their zones of optimal performance, should be explorable (cf. Ellison and Jasienska, 2007). This exploration is not helped, and indeed is often hindered by, use of the term “stress.” For plant ecologists and evolutionary biologists interested in discovering how the plant form-function relationship has evolved, value-laden conceptions of “stress” can be replaced by biologically rich methodological approaches such as those shown in **Figure 2B**. Clearly, the plants in “stressed” (high altitude) habitats grow slowly. Study of vital proportionalities between parts from the point of view that we suggest, however, reveals that these plants are probably well acclimated to the local conditions (**Figure 2C**). Use of the term “stress” only masks this adaptive adjustment. Whatever the causes of variation in allometric slopes or intercepts, the allometric perspective we describe here offers a means for thinking about “stress” in quantitative terms. This framework will allow exploring simultaneously adaptation, acclimation, and “stress” in plants in a quantitative way beyond artificial categorization of these concepts (see Lortie et al., 2004), and will thus serve as a basis for testing a wide array of hypotheses regarding plant performance and fitness.

## Author Contributions

TA, GP, and MO developed the idea, provided the main experimental data, wrote the first draft of the manuscript and revised the text, SL provided additional experimental data, FS and SL interpreted the data and intensively discussed and revised the text.

## Conflict of Interest Statement

The authors declare that the research was conducted in the absence of any commercial or financial relationships that could be construed as a potential conflict of interest.
